# Role of CD151, A tetraspanin, in porcine reproductive and respiratory syndrome virus infection

**DOI:** 10.1186/1743-422X-4-62

**Published:** 2007-06-16

**Authors:** Kumar Shanmukhappa, Jeong-Ki Kim, Sanjay Kapil

**Affiliations:** 1 Division of Pediatric Gastroenterology, Hepatology and Nutrition, Cincinnati Children's Hospital Medical Center, University of Cincinnati. Cincinnati, OH 42229, USA; 2 Division of Virology, Department of Infectious Diseases, St. Jude Children's Research Hospital, Memphis, TN 38105, USA; 3 Oklahoma Animal Disease Diagnostic Laboratory, Center for Veterinary Health Sciences, Oklahoma State University, Stillwater, OK 74078, USA

## Abstract

**Background:**

Porcine reproductive and respiratory syndrome virus (PRRSV) is a RNA virus causing respiratory and reproductive diseases in swine. The susceptibility for PRRSV varies between the different breeds of swine. In cell culture, PRRSV virus can be propagated in primary porcine alveolar macrophages and some African green monkey kidney cell lines, such as MARC-145 cells. Previous studies have shown that 3' untranslated region (UTR) RNAs of the arteriviruses play an important role in the replication of the virus through interactions with cellular proteins. To better understand the differences in the replication capability of PRRSV in different cell lines, we sought to identify the host cellular proteins interacting with PRRSV 3' UTR RNA. We constructed a cDNA library of MARC-145 cell line in lambda ZAP Express vector and screened the library with the positive sense 3' UTR RNA of PRRSV.

**Results:**

We found that CD151, a host cellular protein, interacting with PRRSV 3' UTR RNA. The specificity of the interaction between CD151 and PRRSV 3' UTR RNA was examined by gel shift assay as well as North-Western hybridization. The transfection of CD151 expression clone into BHK-21 rendered these cells susceptible to PRRSV infection, and the transfection of siRNA against CD151 into MARC-145 significantly reduced the level of PRRSV infection. Also, anti-CD151 antibody treatment to MARC-145 completely blocked PRRSV infection.

**Conclusion:**

Based on our results, we suggest that CD151 should cooperate in PRRSV infection *in vitro *in MARC-145 and BHK-21 cells.

## Background

Porcine reproductive and respiratory syndrome virus (PRRSV) is the causative agent of viral disease in swine that is endemic in swine producing regions throughout the world resulting in severe economic losses in affected areas. The disease is characterized by severe reproductive failure in sows and gilts and respiratory distress in pigs of all ages [1-3]. PRRSV is an enveloped virus containing single-stranded positive-sense RNA as the genome. Its genome is 14.5 kb in length and is composed of nine open reading frames (ORFs; ORF 1a, ORF 1b, ORF 2a, ORF 2b, ORF 3, ORF 4, ORF 5, ORF 6 and ORF 7) flanked by 5' and 3' untranslated regions (UTRs) [4,5]. PRRSV belongs to the family *Arteriviridae*, grouped together with the *Coronaviridae *and *Roniviridae *in the order *Nidovirales *[6-8]. Other members in the family *Arteriviridae *include equine arteritis virus, lactate dehydrogenase-elevating virus of mice, and simian hemorrhagic fever virus [9].

PRRSV has a restricted cell tropism in its host (pig). It primarily infects alveolar macrophages although the virus has been detected in macrophages of other tissues like spleen, liver, peyers patches, thymus as well as microglial cells, however peritoneal macrophages are refractory [10,11]. Primary cultures of porcine alveolar macrophages are routinely used for *in vitro *isolation of PRRSV. Other established cell lines such as MA104 (a monkey kidney cell line) or its derivatives MARC-145 and CL2621 cells are commonly used for its *in vitro *propagation [2,12]. Several candidate molecules have been identified to be the receptors/co-receptors for PRRSV entry including heparin sulfate and sialoadhesin [13-15]. Our laboratory has demonstrated that PRRSV utilizes vimentin as a receptor in MARC-145 cells [16]. During infection, PRRSV enters the host cells by a receptor-mediated endocytosis through interaction with its receptor(s) and/or co-receptor(s) [17,18]. There are few other cell lines that supports binding of PRRSV but are not permissive to virus infection. Following receptor mediated endocytosis, PRRSV replication proceeds by discontinuous transcription forming a 3'-coterminal nested set of functionally monocistronic mRNA. The common leader sequences in mRNA are joined to the coding sequences by consensus intergenic sequences through the junction sequence UCAACC. The interactions between the leader sequence, the intergenic sequence, and the body of RNA are regulated by *cis- *and *trans*-acting elements as well as host cellular factors [4,5,19,20]. In several RNA viruses, the secondary or tertiary structures of 5' and/or 3' UTRs have been reported to be critical for the viral replication process. In this process, host cellular proteins are thought to bind to 3' UTR of viral RNA [8,20-23]. For example, translation elongation factor 1 alpha was found to bind to the 3' UTR RNAs of West Nile virus [24], dengue virus [25], and tobacco mosaic virus [26]. In corona viruses, Mitochondrial heat shock proteins (hsp 40, 60 and 70) were reported to bind to the 3' UTR RNA of murine hepatitis virus in cooperation with mitochondrial aconitase [27,28]. Recently, glyceraldehyde-3-phosphate dehydrogenase was also reported to interact with hepatitis A virus RNA [29]. These studies indicate that host proteins interacting with 3' UTR RNA of viruses play a very important role in viral infection. Previous studies in our laboratory have identified at least 11 MARC-145 cellular proteins that bind to the 3' UTR RNA of PRRSV (Fahad and Kapil, unpublished data). We performed this study with the aim of identifying these cellular proteins interacting with 3' UTR RNA of PRRSV and to study their role in viral infection.

In this study, we identified a PRRSV 3' UTR RNA-binding protein, CD151, by RNA-ligand screening of a MARC-145 cell expression library. CD151 is a member of the tetraspanin superfamily, which has several cellular functions that include cell signaling, cell activation and platelet aggregation [30-33]. Transfection of CD151 rendered BHK-21, a non-susceptible cell line, susceptible to PRRSV infection. The transfection of siRNA against CD151 inhibited PRRSV infection into MARC-145 cells. Additionally, polyclonal anti-CD151 antibody (Ab) completely blocked PRRSV infection into MARC-145 cells. These results suggest that CD151 plays a critical role in PRRSV infection *in vitro*.

## Results

### Identification of PRRSV 3' UTR RNA-binding clone

**Figure 1:**
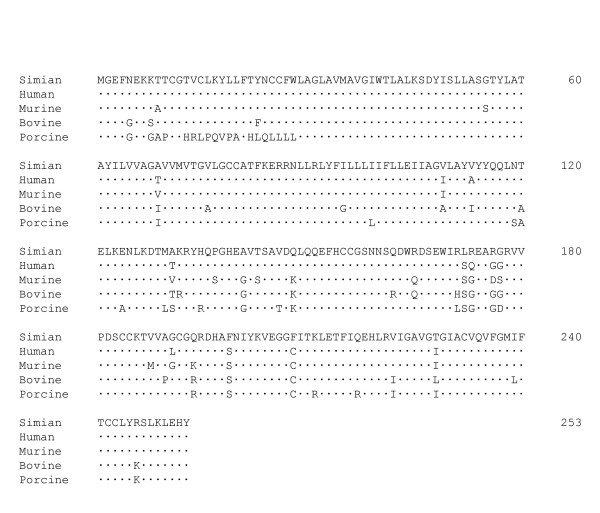
**Alignment of CD151 amino acid sequences**. Simian CD151 amino acid sequence was generated from the cDNA sequence. The amino acid sequence was aligned with human, bovine, murine and porcine CD151 amino acid sequences. Dots represent similarity of amino acid residues. Genbank accession number is AF 275666 [Genbank: AF275666].

To identify the host cellular proteins binding to 3' UTR of PRRSV, we constructed a MARC-145 cell line cDNA library in our laboratory. The library had a titer of 10^8 ^plaque forming units/ml with an average insert size of 1–4 kb (data not shown). The library was screened by North-Western hybridization using α-^32^P-labeled 3' UTR RNA of PRRSV. Approximately 6 × 10^6 ^plaques were screened, and a single reacting clone was obtained by repeated plaque purification and re-screening five times (data not shown). In the last round of screening, a single plaque was isolated, rescued and sequenced. The insert was identified as CD151 by NCBI BLAST search. Figure 1 shows the alignment of the simian CD151 amino acid sequence (Genebank accession number: AF275666) with human, bovine, murine, and porcine CD151 amino acid sequences. The simian CD151 amino acid sequence has 95%, 92%, 89% and 83% identity with human, bovine, murine and porcine CD151 amino acid sequences respectively.

### *In vitro *binding activity of simian CD151 to PRRSV 3' UTR RNA

**Figure 2:**
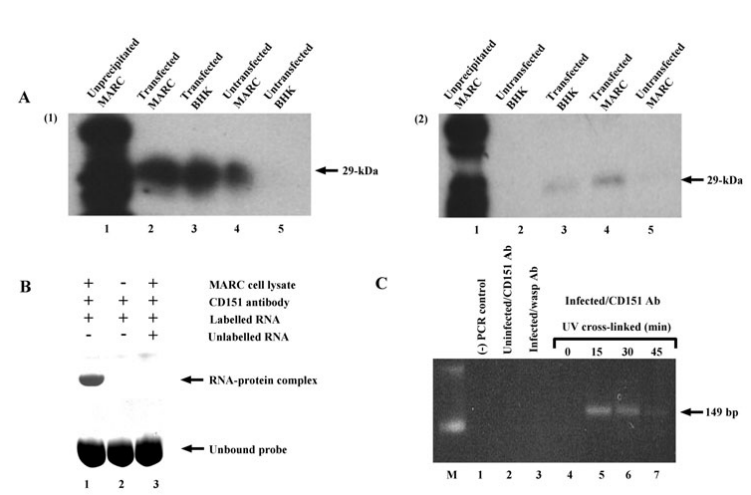
**RNA-binding activity of CD151 *in vitro *and *in vivo***. (A) *In vito *RNA-binding activity of CD151 was demonstrated by Immunoprecipitation/North-Western blot analysis. BHK-21 and MARC-145 cells were transfected with pBK-CMV plasmid expressing CD 151 as a β-galactosidase fusion protein. The cell lysates were immunoprecipitated with anti-CD151 MAb (A1) and anti-β-galactosidase MAb (A2). In A1, lane1, MARC-145 cytoplasmic protein lysate (without immunoprecipitation); lanes 2, transfected MARC-145; lane 3, transfected BHK-21; lane 4, untransfected MARC-145; lane 5, untransfected BHK-21. In A (2), lane1, MARC-145 cytoplasmic protein lysate (without immunoprecipitation); lane 2, untransfected BHK-21; lane 3, transfected BHK-21; lane 4, transfected MARC-145; lane 5, untransfected MARC-145. FIG 2B, gel shift assay demonstrating the interaction of CD151 protein with the PRRSV 3'UTR RNA. MARC cell lysate was immunoprecipitated with CD151 antibody (lanes 1&3) and the complex was incubated radiolabelled PRRSV 3' UTR RNA. Addition of unlabelled RNA (lane 3) prevented the formation of complex, while the radiolabelled RNA did not interact with the CD151 antibody (lane 3). FIG 2C, *In vivo *RNA-binding activity of CD151 was demonstrated by immunoprecipitation/RT-PCR assay (149 bp amplicon). PRRSV-infected or uninfected MARC-145 cell lysates were immunoprecipitated with anti-CD151 MAb or a negative control MAb (wasp, *Cotesia folepis *MAb), and RT-PCR was performed using PRRSV 3' UTR RNA-specific primers for RNAs extracted from the immunocomplexes. M, 123 bp ladder; lane 1, negative PCR control; lane 2, PRRSV-uninfected/CD151 MAb-immunoprecipitated; lane 3, PRRSV-infected/wasp MAb-immunoprecipitated; lane 4, PRRSV-infected/CD151 MAb-immunoprecipitated (without UV cross-linking); lane 5, PRRSV-infected/CD151 MAb-immunoprecipitated (UV cross-linked for 15 min); lane 6, PRRSV-infected/CD151 MAb-immunoprecipitated (UV cross-linked for 30 min); lane 7, PRRSV-infected/CD151 MAb-immunoprecipitated (UV cross-linked for 45 min).

North-Western hybridization was performed to demonstrate the interaction between CD151 protein and PRRSV 3' UTR RNA. MARC-145 and BHK-21 cells were transfected with CD151 plasmid isolated from the cDNA library screened and the protein was immunoprecipitated with anti-CD151 Ab. Then, the immunocomplex was electrophoresed by SDS-PAGE, and the RNA-binding activity was detected by North-Western hybridization using α-^32^P-labeled PRRSV 3' UTR RNA probe. Figure 2A (1) demonstrates the RNA-binding activity of the CD151 protein in CD151-transfected MARC-145 {Fig. 2A (1), lane 2} or BHK-21 cell lysates {Fig. 2A (1), lane 3}. The endogenous CD151 also has PRRSV 3' UTR RNA-binding activity (untransfected MARC-145 cell lysates Fig. 2A, lane 4). However the untransfected BHK-21 cells did not demonstrate any RNA binding activity as these cells lack CD151 protein.

Since simian CD151 was expressed as a lac Z fusion protein, simian CD151-transfected or untransfected cell lysates were also immunoprecipitated with anti-β-galactosidase MAb. Fig. 2A (2) shows PRRSV 3' UTR RNA-binding activity of the immunocomplex immunoprecipitated with anti-β-galactosidase MAb from simian CD151-transfected BHK-21 {Fig. 2A (2), lane 3} or MARC-145 cell lysates; {Fig. 2A (2), lane 4}. However, the immunocomplex immunoprecipitated with anti-β-galactosidase MAb from untransfected BHK-21 {Fig. 2A (2), lane 2} or MARC-145 cell lysates {Fig. 2A (2), lane 5} did not show PRRSV 3' UTR RNA-binding activity.

To directly demonstrate the interaction between CD151 and PRRSV 3' UTR RNA, we performed gel shift assay {Fig 2B lane 1}. Upon addition of the cold, unlabelled RNA, we found that the interaction was inhibited {lane 3} nor did the PRRSV RNA interact with CD151 antibody {lane 2}. These results indicate that CD151 interacts specifically with PRRSV 3' UTR RNA.

### *In vivo *binding activity of simian CD151 to PRRSV 3' UTR RNA

After demonstrating that CD151 protein interacts with PRRSV 3' UTR RNA *in vitro*, we wanted to determine if the interaction also occurs *in vivo*. It has been demonstrated earlier that UV cross-linking strengthens and preserve RNA-protein complexes that also withstands immunprecipitation [34-38]. MARC-145 cells were infected with PRRSV, and after UV cross-linking, the cytoplasmic proteins were isolated and immunoprecipitated with anti-CD151 MAb. Then, RNA was isolated from the immunocomplex, and RT-PCR was performed using PRRSV 3' UTR RNA-specific primers. PRRSV 3' UTR was detected in the immunocomplex demonstrating that the CD151 protein interacts *in vivo *with PRRSV 3' UTR. {Fig. 2C, lane 4–7}. However, PRRSV 3' UTR RNA was neither detected in the immunocomplex from uninfected MARC-145 cells using anti-CD151 MAb {Fig. 2C, lane 2} nor detected in the immunocomplex from PRRSV-infected MARC-145 cells using the isotype control MAb against wasp protein Cotesia folepis. {Fig. 2C, lane 3}. These results clearly demonstrate that CD151 protein interacts with 3' UTR RNA of PRRSV.

### Correlation between CD151 expression and susceptibility to PRRSV infection

**Figure 3:**
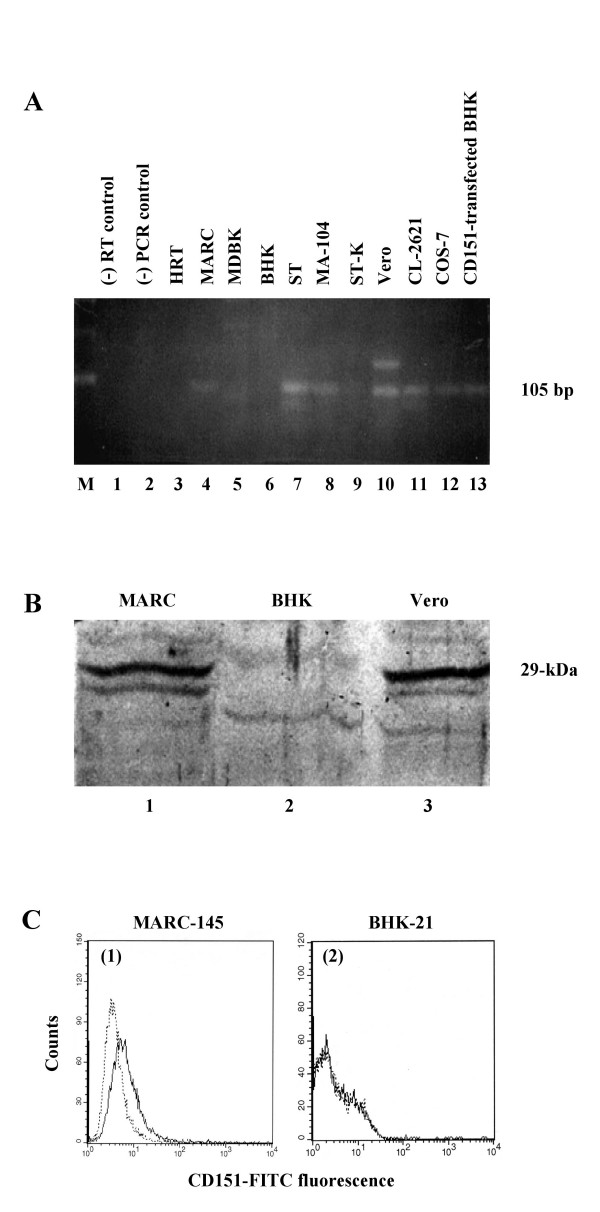
**Detection of the presence of CD151 by RT-PCR and Western blot**. Correlation between CD151 expression and susceptibility to PRRSV infection was demonstrated by RT-PCR and Western blot analysis. (A) RT-PCR showing the amplification of 105 bp amplicon with CD151-specific primers was performed for RNAs isolated from PRRSV-susceptible and -non-susceptible cell lines. M, 123 bp ladder; lane 1, negative RT control; lane 2, negative PCR control; lane 3, HRT; lane 4, MARC-145; lane 5, MDBK; lane 6, BHK-21; lane 7, ST; lane 8, MA-104; lane 9, ST-K; lane 10, Vero; lane 11, CL-2621; lane 12, COS; lane 13, CD151-transfected BHK-21. (B) Western blot analysis using anti-CD151 MAb was performed for cell lysates from PRRSV-susceptible and -non susceptible cell lines. Lane 1, MARC-145; lane 2, BHK-21; lane 3, Vero. (C) Flow cytometric analysis using polyclonal anti-CD151 Ab was performed for MARC-145 (C (1)) and BHK-21 (C (2)) cell lines. An isotype-matched control is represented by the dotted lines.

To determine the possible relationship between the presence of CD151 and susceptibility to PRRSV infection, we screened various PRRSV susceptible and non-susceptible cell lines using RT-PCR for CD151. As shown in Fig. 3A, a 105 bp amplicon of CD151 was amplified in MARC-145 {Fig. 3A, lane 4}, ST {Fig. 3A, lane 7}, MA-104 {Fig. 3A, lane 8}, ST-K {Fig. 3A, lane 9}, Vero {Fig. 3A, lane 10}, CL-2621 {Fig. 3A, lane 11}, COS-7 {Fig. 3A, lane 12}, and simian CD151-transfected BHK-21 cells {Fig. 3A, lane 13}. However, the 105 bp amplicon of CD151 was not amplified in HRT {Fig. 3A, lane 3}, MDBK {Fig. 3A, lane 5} and BHK-21 cells {Fig. 3A, lane 6}. MARC-145, MA-104, CL-2621 and Vero cells are known to be susceptible to PRRSV infection, while BHK-21 cells are known to be non-susceptible [19,20]. We also performed Western blot analysis using anti-CD151 MAb to determine the presence of CD151 in some of the PRRSV-susceptible and -non-susceptible cell lines. As shown in Figure 3B, CD151 was detected in susceptible cell lines, MARC-145 {Fig. 3B, lane 1} and Vero {Fig. 3B, lane 3}, while CD151 was not detected in a non-susceptible cell line, BHK-21 {Fig. 3B, lane 2}. Additionally, we also found the expression of CD151 protein by flow cytometric analysis in MARC-145 and BHK-21 cells. CD151 protein was expressed on the surface of MARC 145 cells but not on surface of BHK-21 cells {Fig 3C}.

### Transfection of non-susceptible cell line (BHK-21) with CD151 confers susceptibility to PRRSV

**Figure 4:**
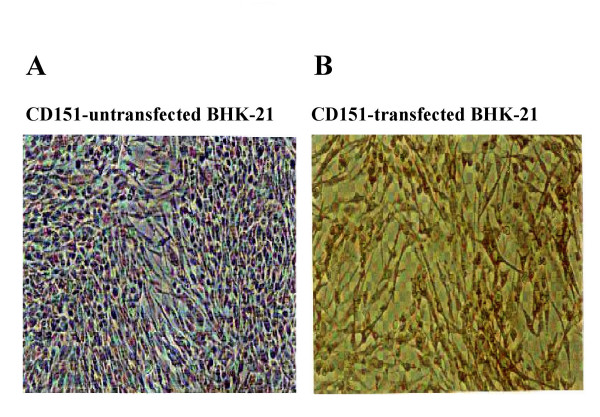
**Transfection of simian CD151 into BHK-21 cells**. To detect the presence of PRRSV in simian CD151-transfected BHK-21 cells, immunohistochemical staining was performed using SR-30, a MAb against PRRSV nucleocapsid protein. (A) Simian CD151-untransfected BHK-21 cells, and (B) Simian CD151-transfected BHK-21 cells. The presence of PRRSV is shown by DAB substrate in brown color.

The PRRSV non-susceptible cell line, BHK-21 was transfected with the pBK-CMV plasmid containing CD151 gene and then was infected with PRRSV. Immunohistochemical staining was performed to detect the presence of PRRSV in simian CD151-transfected BHK-21 cells using SR-30, a MAb against PRRSV nucleocapsid protein. As shown in Fig. 4, CD151-transfected BHK-21 cells could be infected with PRRSV {Fig. 4A}, while untransfected BHK-21 cells could not be infected with PRRSV {Fig. 4B}. Where as the BHK-21 cells transfected with control plasmid (CMV driven β-gal protein) did not confer susceptibility to PRRSV infection (data not shown). These results indicate that CD151 should be one of the susceptibility factors to PRRSV infection.

### Interaction between CD151 and PRRSV proteins

The interaction between CD151 and PRRSV proteins and CD151 was investigated by (co-) immunoprecipitation. The infected MARC-145 cells were immunoprecipitated with anti-CD151 MAb, and the presence of PRRSV proteins in the immunocomplex was examined by PRRSV hyperimmune serum, followed by detection with the ECL system. The co-immunoprecipitation was also performed by immunoprecipitating with PRRSV hyperimmune serum, and the presence of CD151 in the immunocomplex was examined by anti-CD151 MAb. Virus overlay protein binding assay (VOPBA) was performed to investigate if there is any direct interaction between PRRSV proteins and CD151 as described ([39]. However, any direct interactions between the CD151 and PRRSV proteins were not detected (data not shown).

### Effect of CD151-overexpression on PRRSV infection levels

**Figure 5:**
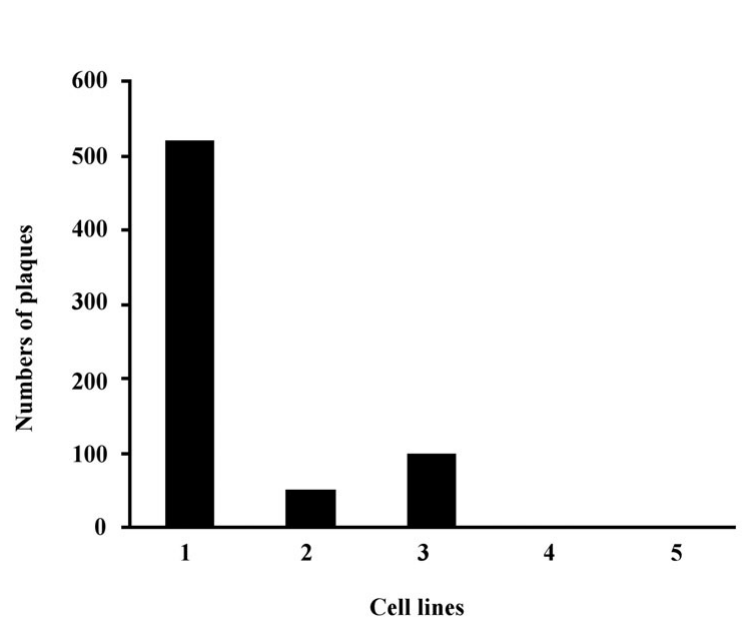
**Effect of CD151-overexpression on PRRSV infection**. The effect of CD151-overexpression on PRRSV infection was demonstrated by virus burst assay. To induce CD151-overexpression, the simian CD151 expressing clone was transfected into MARC-145 cells. Column 1, CD151-transfected/PRRSV-infected MARC-145; column 2, β-galactosidase-transfected/PRRSV-infected MARC-145; column 3, CD151-transfected/PRRSV-infected BHK-21; column 4, CD151-untransfected/PRRSV-infected BHK-21; column 5, CD151-transfected/PRRSV-uninfected MARC.

To address the effect of CD151-overexpression on PRRSV infection, MARC cells were examined with respect to the effect on infectivity level. Both CD151-transfected and untransfected MARC-145 cells were infected with equal amounts of plaque-purified PRRSV. The cells were allowed to grow for one complete replication cycle (18 hr), and the infectivity levels of PRRSV in both simian CD151-transfected and -untransfected MARC-145 cells were measured by plaque assay. Additionally, simian CD151-transfected BHK-21 cells were also examined. As shown in Fig. 5, there was approximately a 100-fold increase in the amount of virus in the simian CD151-transfected MARC-145 cells overexpressing CD151 {Fig. 5, column 1} as compared to untransfected MARC-145 cells {Fig. 5, column 2}. The simian CD151-transfected BHK-21 cells also allowed for PRRSV replication at a higher level than untransfected MARC-145 cells {Fig. 5, column 3}.

### Effect of siRNA against CD 151

**Figure 6:**
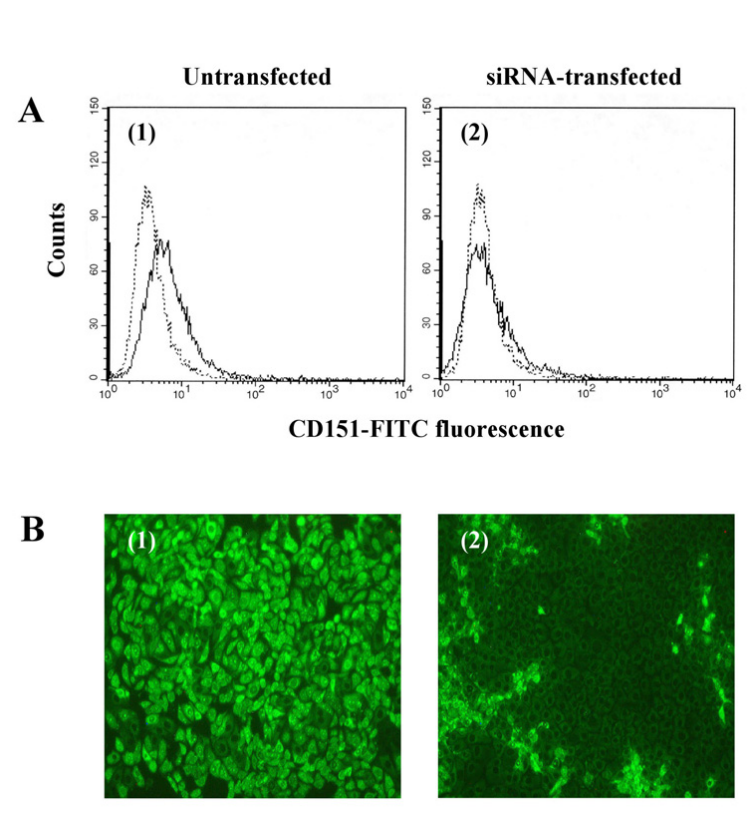
**Effect of siRNA against CD151 on PRRSV infection**. (A) To examine the effect of siRNA against CD151 on PRRSV infection, siRNA was transfected into MARC-145 cells. The suppression of the cell surface expression of CD151 by the transfection of siRNA was shown by flow cytometric analysis for the untransfected MARC-145 cells (A1) and the transfected MARC-145 cells (A2). An isotype-matched control is represented by the dotted lines. (B) The effect of siRNA on PRRSV infection was shown by immunofluorescence antibody assay using FITC-conjugated SDOW-17, a MAb against PRRSV nucleocapsid protein for the untransfected MARC-145 cells (B 1) and the transfected MARC-145 cells (B 2).

To study the effect of suppression of CD151 expression on PRRSV replication, the transfection of siRNA against CD151 was performed with MARC-145 cells. Figure 6A shows the effect of the transfection of siRNA against CD151 on CD151 expression. The expression level of CD151 was reduced (36% to 19%) by the transfection of siRNA against CD151 {Fig. 6A (2)}, even though the expression level of CD151 in the mock-transfected MARC-145 cells was not high {Fig. 6A (1)}. Figure 6B shows the effect of the transfection of siRNA against CD151 on PRRSV infection. PRRSV infection was significantly reduced (50% reduction as determined by fluorescent staining) by the transfection of siRNA against CD151 {Fig. 6B (2)}.

### Blocking activity of anti-CD151 Ab on PRRSV infection into MARC-145 cells

**Figure 7:**
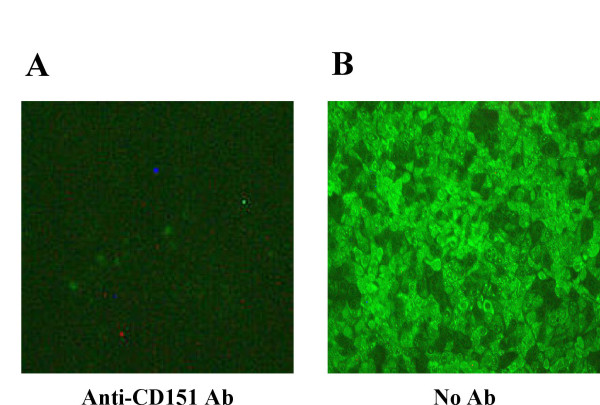
**Effect of anti-CD151 Ab on PRRSV infection**. To examine the effect of anti-CD151 Ab on PRRSV infection, immunofluorescence antibody assay was performed. MARC-145 cells were incubated with polyclonal anti-CD151 Ab (A) or PBS (B) and infected with PRRSV. At 2 days post infection, the presence of PRRSV in the cells was detected by FITC-conjugated SDOW-17, a MAb against PRRSV nucleocapsid protein.

To investigate the effect of polyclonal anti-CD151 Ab on PRRSV infection into MARC-145 cells, a checkerboard titration assay was performed. As shown in Table 1, polyclonal anti-CD151 Ab blocked PRRSV infection in a dose-dependent manner. Even at the highest concentration of the virus (10^-1^-dilution), polyclonal anti-CD151 Ab completely blocked PRRSV infection. However, a negative control Ab, anti-β-galactosidase MAb, did not block PRRSV infection (data not shown). Figure 7 shows the complete blocking activity of polyclonal anti-CD151 Ab on PRRSV infection by immunofluorescence antibody assay.

**Table 1:** Checkerboard titration assay for measuring the blocking activity of anti-CD151 Ab

**Virus dilution (1:9-diluted) ↓**	10^-1^	C	C	C	0	0	1	2.5	3	3	3	3	3
		
	10^-2^	C	c	c	0	0	1	2.5	3	3	3	3	3
	10^-3^	C	c	c	0	0	0.5	1	2	2	2	2	2
	10^-4^	c	c	c	0	0	0	0	0.5	1	1	1	1
	10^-5^	c	c	c	0	0	0	0	0	0.5	0.5	0.5	0.5
	10^-6^	c	c	c	0	0	0	0	0	0	0	0	0
	10^-7^	c	c	c	0	0	0	0	0	0	0	0	0
		
	**No virus**	C	c	c	0	0	0	0	0	0	0	0	0
		
		**Ab dilution(1:1-diluted) →**									**No Ab**		
		20^-1^	40^-1^	80^-1^	160^-1^	320^-1^	640^-1^	1280^-1^	2560^-1^	5120^-1^			

MARC-145 cells were cultured with polyclonal anti-CD151 Ab and/or PRRSV in a 96-well tissue culture plate. Polyclonal anti-CD151 Ab was 1:1-serially diluted from 20^-1^-dilution, and the PRRSV preparation was 1:9-serially diluted from 10^-1^-dilution. At 2 days post infection, immunofluorescence microscopy analysis was performed. The cells were stained with FITC-conjugated SDOW-17, a MAb against PRRSV nucleocapsid protein. The cells were examined by fluorescent microscopy. C means the cytopathic effect of Ab, and the numbers mean the intensity of fluorescence (0 means no fluorescence detected, and 3 means the highest intensity of fluorescence)

## Discussion

Viruses are obligate intracellular parasites, which use host cellular factors and energy supplies for replication. In several RNA viruses, the interaction between 5' and/or 3' UTR RNA and host cell proteins was already reported to play an important role in virus replication mechanisms, such as the transcription, translation, orientation and transport of viral RNA [23,40].

In this study we were able to demonstrate for the first time that CD151 protein binds to 3' UTR RNA of PRRSV. Interaction between CD151 and RNA of PRRSV is specific (Gel shift assay) and interaction also occurs *in vivo *(detection of PRRSV RNA in immunoprecipitation). Another important observation of our study is that CD151 confers PRRSV susceptibility to BHK-21 cells. Previously it has been shown that BHK-21 cells are non-susceptible to PRRSV infection. However these cells when transfected with either PRRSV RNA or infectious cDNA clones, it results in productive infection of PRRSV without spreading to neighbouring cells [19]. The major factor that is lacking in BHK-21 cells that prevent the infection seems to be in entry. Since CD151 is a transmembrane protein, we reasoned that it might function as the entry molecule and performed (co-) immunoprecipitation experiments to determine if there is direct interaction between CD151 and the PRRSV protein. We could not detect any direct interaction between them using (co-) immunoprecipitation and virus overlay protein binding assay (data not shown). Our results are in agreement with role of another tetraspanin molecule CD9 that has been shown to render MDBK cells susceptible to infection by a canine distemper virus (CDV) and predicted that this molecule serves as the entry molecule. However, they also could not demonstrate any direct interaction between CD9 and CDV proteins [40]. Therefore we cannot completely rule out the possibility of interaction between the CD151 and PRRSV proteins leading to helping of virus entry into BHK-21 cells.

CD151 is a 29-kDa transmembrane glycoprotein with an N-glycosylation site and several palmitoylation sites [41,42]. CD151 is a member of the tetraspanin superfamily, alternately known as the transmembrane 4 superfamily, which is characterized by the presence of four highly conserved hydrophobic transmembrane domains. CD151 was initially identified as a human platelet surface glycoprotein (platelet endothelial tetraspan antigen-3; PETA-3) by a monoclonal antibody inducing platelet aggregation [43]. CD151 was also independently cloned as SF-HT-activated gene 1 (SFA-1), which was up-regulated in human T cells by transformation with human T-cell-leukemia virus type 1 [44]. We found that CD151 protein is highly conserved across the species examined with high homology between human and simian species and our results are in agreement with previous report [45]. In this study, we examined the expression of CD151 in several cell lines to determine if it is the susceptibility factor in PRRSV infection. CD151 was expressed in all susceptible cell lines namely, MA-104, MARC-145, COS-7 and Vero cells, which are derived from African green monkey kidney. However, CD151 was not expressed in BHK-21 and MDBK cells, which are derived from kidneys of the other species. CD151 has a wide cell and tissue distribution, including platelets, megakaryocytes, activated T lymphocytes, dendritic cells, Schwann cells, epithelial cells, endothelial cells, and muscle cells [43,44,46]. In account of our novel observation of RNA binding activity of CD151, we looked for RNA binding domains on CD151 protein by bioinformatic analysis, we could not find any known RNA binding activity but there were some motifs in second extracellular domain which could be potential RNA binding sites. Current experiments are underway to identify potential RNA binding motifs.

Evidence presented in this study definitely points that CD151 confers susceptibility to PRRSV infection. It is evident when transfection of a CD151 expressing clone into MARC-145 cells increased the susceptibility of MARC cells to PRRSV. Conversely, decreased expression of CD151 by using siRNA also inhibited the susceptibility of MARC-145 cells to PRRSV infection. Furthermore, the antibody against CD151 completely inhibited PRRSV infection of MARC-145 cells. These results indicate that CD151 plays very important role in PRRSV infection of MARC-145 cells. To this end, only direct interaction between CD151 and PRRSV is that of RNA-protein interaction. How can CD151, a transmembarane protein, by virtue of its binding to PRRSV RNA help in virus infection? PRRSV and other arteriviruses, enter into host cells by receptor-mediated endocytosis. CD151, by virtue of its expression on the plasma membranes and in intracellular vesicles, like endosomes [33,46], interacts with PRRSV in cooperation with other molecules [13-18]. Even though we could not directly demonstrate the interaction between CD151 and PRRSV protein, we cannot rule if there is any direct interaction between them. Another example of tetraspan molecule promoting viral entry is CD82 and CD81 molecules in case of HTLV-1 virus [47-49], however in this case, binding of CD81 to viral glycoprotein E2 does not correlate with permissiveness of cells to virus infection. This implies that other cellular factors are required for viral infection [47-49]. During endocytosis, lowering of pH in the endosome results in fusion event between viral envelope and endosome [18] possibly involving CD151. Another role of CD151 by virtue of RNA binding ability is possibly in localization of ribonucleoprotein complexes to the site of viral replication [21,41] that has been demonstrated to promote viral replication.

## Conclusion

Based on our results, we propose that CD151 is one of the key molecule in facilitating PRRSV infection. To our knowledge, it is the first demonstration of the interaction between PRRSV 3' UTR RNA and a host cell protein, CD151.

## Methods

### Cell lines and virus

African green monkey kidney cell lines (MARC-145, COS-7, Vero, CL-2621 and MA-104), a baby hamster kidney cell line (BHK-21), a bovine kidney cell line (MDBK), a swine testis cell line (ST) and a human rectal tumor cell line (HRT) were used in the study. These cell lines obtained from ATCC were already available in our laboratory. The cell lines were grown in Eagle's minimum essential medium (MEM; Life Technologies, Inc., Gaithersburg, MD) supplemented with 10% fetal bovine serum (FBS; Hyclone, Logan, UT). The ATCC VR-2332 strain of PRRSV was used in the study. The virus was propagated in MARC-145 cells.

### Construction of MARC-145 cDNA library

The cDNA library from MARC-145 cells was constructed in our laboratory using a λ ZAP Express cDNA synthesis kit (Stratagene, La Jolla, CA) by following manufacturer's instructions. Briefly, total cellular RNA from MARC-145 cells was extracted according to the Chomczynski and Sacchi method [34]. The mRNA was purified from total cellular RNA using an oligo (dT) cellulose column (Stratagene, La Jolla, CA), and then 5 μg of mRNA was converted to cDNA. The cDNA was then directionally cloned in the λ ZAP Express vector. The cDNA library was packaged using the ZAP Express cDNA Gigapack III Gold cloning kit (Stratagene, La Jolla, CA).

### Cloning of PRRSV 3' UTR RNA and RNA probe preparation

PRRSV 3' UTR was amplified by RT-PCR using forward 5'-CCCCATTTTCCTCTA

GCGACTG-3' and reverse 5'-CGGCCGCATGGTTCTCGCCAAT-3' primers (regions corresponding to 15,386 to 15,846 bp of the PRRSV VR-2332) and then cloned into the pCR II vector (Invitrogen, Carlsbad, CA). α-^32^P-labeled 3' UTR RNA transcript was prepared by *in vitro *transcription using a T7 RNA synthesis kit, Riboscribe™ (Epicentre Technologies, Madison, WI) by following the manufacturer's instructions. The probe was purified either by Quick Spin™ columns (Boehringer Mannheim, Indianapolis, IN) for North-Western blotting or by acrylamide gel electrophoresis [35] method of purification for gel mobility shift assay.

### North-Western screening of MARC-145 cDNA library

The MARC-145 cDNA library was screened using PRRSV 3' UTR RNA by North-Western hybridization described [36]. In all the rounds of the screening, protein expression was induced using nitrocellulose membranes impregnated with 10 mM IPTG for 2 hr. The nitrocellulose membranes were denatured in 6 M guanidinium hydrochloride for 30 min, followed by sequential renaturation every 10 min with equal changes of single-binding (SB) buffer (15 mM HEPES [pH 7.9], 50 mM KCl, 0.01% [vol/vol] Nonidet P-40, 0.1% [wt/vol] Ficoll 400-DL, 0.1% [wt/vol] PVP-40, 0.1 mM MnCl_2, _0.1 mM ZnCl_2, _0.1 mM EDTA and 0.5 mM DTT) for 1 hr. Hybridization was performed in SB buffer containing the α-^32^P-labeled PRRSV 3' UTR RNA probe at 500,000 cpm/ml in presence of 10 μg/ml of yeast tRNA and 100 μg/ml of denatured sheared salmon sperm DNA overnight. The blots were washed with SB buffer for 1.5 hr, and RNA-binding activity was detected by autoradiography. The corresponding positive plaques were cored, eluted and then rescued using the ZAP Express cDNA Gigapack III Gold cloning kit (Stratagene, La Jolla, CA). Sequencing was performed at the Iowa State University Sequencing Facility in Ames, IA.

### Transfection of CD151 clone

BHK-21 and MARC-145 cells were transfected with pBK-CMV plasmid containing CD151 gene using Lipofectamine™ reagent (Life Technologies, Inc., Gaithersburg, MD) by following manufacturer's instructions. For transient transfection, the cells were tested for protein expression 24 hrs after transfection. For stable transfection, media was changed to selection medium containing G418 sulfate (Omega Scientific, Inc., Tarzana, CA) in growth medium (1 mg/ml for BHK-21 cells and 0.7 mg/ml for MARC-145 cells). After selection, the cells were maintained in the presence of G418 sulfate at 0.5 mg/ml for BHK-21 cells and 0.35 mg/ml for MARC-145 cells. The expression of CD151 was measured by immunoprecipitation followed by North-Western hybridization.

### Immunoprecipitation/North-Western hybridization

CD151 protein was immunoprocipitated using anti-CD151 antibody and the RNA binding activity was detected by North-Western hybridization. BHK-21 or MARC-145 cells were transfected with CD151 as described above. The transfected cells were lysed in 1 ml of single detergent lysis buffer (50 mM Tris-HCl [pH8.0], 150 mM NaCl, Phenylmethylsulfonyl fluoride 100 μg/ml and 1% [vol/vol] Nonidet-P40). Proteins were quantified using Bradford method based Bio-Rad assay (Bio-Rad Laboratory Inc., Hercules, CA). To 500 μg of cell lysate, 1 mg/ml of anti-CD 151 MAb (BD Biosciences, Franklin Lakes, NJ) or anti-β-galactosidase MAb (Boehringer Mannheim, Indianapolis, IN) was added and rocked overnight at 4°C. The immunocomplexes were precipitated on ice for 2 h with the addition of 40 μl of protein A-sepharose beads (Sigma, St. Louis, MO) and then centrifuged at 4,000 × *g *for 10 min. The pellets were washed once in cold Tris saline azide (TSA) buffer (0.05 M Tris-HCl [pH 8.0]; 0.15 M NaCl; 0.025% NaN_3_) containing 1% Triton X-100 and 1% SDS. The second wash was done in cold TSA buffer alone, followed by two washes in 10 mM Tris-HCl [pH 7.5] containing 1 mM EDTA. The pellet was suspended in 20 μl of SDS-loading buffer and electrophoresized by SDS-PAGE. The proteins were transferred onto a nitrocellulose membrane, and North-Western hybridization was performed as described above.

### Gel mobility shift assay

To determine the specificity of interaction between CD151 protein and the PRRSV 3' UTR RNA, we performed gel mobility shift assay as described [25] with slight modifications. 500 μg of MARC cell lysate was immunoprecipitated with anti-CD151 MAb as described above. After washing the immunocomplexes, the immunoprecipitate was resuspended in 50 μl of incubation buffer (50 mM HEPES [pH7.4], 0.1 mM DTT, 40 mM MgCl_2_, 0.5 mM EDTA, 20 mM Spermidine, 1.5 mM ATP, 10 mM GTP) along with 4 μg of yeast tRNA and incubated for 10 min at 4°C. Labeled RNA (500,000 cpm) was added and incubated further for 15 min. For competition experiments, unlabelled RNA (3 fold excess) was included in the pre-incubation prior to addition of labeled RNA.

### *In vivo *cross-linking and reverse transcription (RT)-PCR assay

To investigate *in vivo *interaction between CD151 and PRRSV 3' UTR RNA, *In vivo *cross-linking followed by immunoprecipitation and then RT-PCR was performed as described with slight modifications [37,38] ([39]. MARC-145 cells were infected with PRRSV at 37°C for 1 hr. The cells were washed 3 times in PBS and twice in MEM, and replaced with MEM supplemented with 1% FBS. At 18 hr postinfection, the cells were washed twice in PBS and covered in PBS. Irradiation was performed on ice in a UV cross-linker (Fisher Scientific, Pittsburgh, PA) at a distance of 10 cm from the 300 λ light-source for 0, 15, 30 and 45 min. PBS was removed, and the cells were lysed by adding ice cold RIPA lysis buffer (20 mM Tris-HCl [pH8.0], 150 mM NaCl, 1% Nonidet P-40, 1% SDS and 0.5% deoxycholic acid) supplemented with 20 U of DNase and 20 U of RNasin inhibitors (Life Technologies, Inc., Gaithersburg, MD). Immunoprecipitation was performed using anti-CD151 MAb as described above, except that RNase inhibitor (20 U) was added in all incubations. Immunoprecipitate was treated with Proteinase K (4 μg/ml) at 37°C for 15 min, and RNA was extracted as described previously [34]. To determine the presence of PRRSV 3' UTR RNA, RT-PCR was performed as described below. To detect PRRSV 3' UTR RNA bound to the immunocomplex in *In vivo *cross-linking and RT-PCR assay, RT-PCR was performed using the GeneAmp EZ r*Tth *RNA PCR kit (Roche Molecular System, Inc., Branchburg, NJ) with PRRSV 3' UTR RNA-specific primers; 5'-TGGGCTGGCATTCTTGAGGC-3' (forward) and 5'-TTCGGGCCGCATGGTTCTCGC-3' (reverse) that cover 15,262 bp to 15,410 bp regions of PRRSV VR-2332 strain. Reverse transcription was performed at 42°C for 45 min, 95°C for 10 min and 5°C for 5 min. Standard PCR was done at 95°C for 2 min, 95°C for 30 s, 55°C for 30 s, 72°C for 60 s for 25 cycles and 72°C for 30 min. To demonstrate the correlation between CD151 presence and susceptibility to PRRSV infection, RT-PCR was carried out using CD151 specific primers 5'-CCTACCTGGCCACAGCCTAC-3' (forward) and 5'-ACAGGCGCAGCAGGTTCCGA-3' (reverse) that amplifies 167 bp to 277 bp region of CD151. RNA was isolated from PRRSV-susceptible and non-susceptible cell lines as described previously [34]. Reverse transcription reaction was performed at 42°C for 45 min, 95°C for 10 min and 5°C for 5 min. Standard PCR was done at 95°C for 2 min, 95°C for 30 s, 55°C for 30 s, 72°C for 15 s for 25 cycles and 72°C for 30 min. The PCR products were detected by agarose gel electrophoresis.

### Western blot analysis

To examine the presence of CD151 in MARC-145, BHK-21 and Vero cells, Western blot analysis was performed. MARC-145, BHK-21 and Vero cytoplasmic proteins were electrophoresed by SDS-PAGE and transferred onto a nitrocellulose membrane. After blocking in 5% skim-milk in PBS, the membrane was stained with anti-CD151 MAb at room temperature for 1 hr, followed by staining with the peroxidase-conjugated horse anti-mouse IgG (H+L) (Vector Laboratories, Inc., Burlingame, CA) at room temperature for 45 min. The proteins were detected by the enhanced chemiluminescence (ECL) system (Amersham Biosciences, Piscataway, NJ) by following manufacturer's instructions.

### Flow cytometric analysis

To investigate the cell surface expression of CD151 and quantify CD151 protein in MARC-145 and BHK-21 cells, flow cytometry was performed. After trypsinization, cells (5 × 10^5 ^total) were washed twice in staining solution (0.1% bovine serum albumin [BSA] in PBS) and blocked in 3% BSA in staining solution on ice for 10 min, and then incubated with polyclonal goat anti-CD151 Ab (Santa Cruz Biotechnology, Inc., Santa Cruz, CA) on ice for 30 min. After washing twice in staining solution, cells were incubated with rabbit anti-goat FITC conjugated secondary Ab (Bethyl Laboratories, Montgomery, TX) on ice for 30 min. Cells were resuspended in 1% paraformalehyde in PBS after washing twice in staining solution. Flow cytometric analysis was performed on a FACSCalibur (BD Biosciences, San Jose, CA). In transfection experiment involving siRNA against CD151, the siRNA-transfected MARC-145 cells were stained as described above.

### Immunohistochemistry

To determine if the CD151-transfected BHK-21 cells become susceptible to PRRSV infection, immunohistochemical staining was performed using a MAb against PRRSV nucleocapsid protein. The cells were cultured in a 24 well plate and infected with PRRSV. At 24 hr post infection, the cells were fixed in 75% acetone in PBS at 4°C for 10 min and stained with SR-30 (Rural Technologies, Inc., Brookings, SD), a MAb against PRRSV nucleocapsid protein at 37°C for 1 h, followed by staining with a biotinylated anti-mouse IgG (Vector Labs, Burlingame, CA) at RT for 30 min. Finally, the avidin-biotin-enzyme complex (Vector Labs, Burlingame, CA) was added. The presence of PRRSV in the cells was detected by the addition of DAB substrate (Vector Labs, Burlingame, CA). The cells were counterstained with Gill's-1 hematoxylin and examined by light microscopy.

### Immunoprecipitation/co-immunoprecipitation

To examine the interaction between CD151 and PRRSV proteins, immunoprecipitation was performed. MARC-145 cells were infected with PRRSV, and the cell lysate was prepared in single detergent lysis buffer 2 days post infection. The PRRSV-infected MARC-145 cell lysate was immunoprecipitated with anti-CD151 MAb as described above. The immunocomplex was electrophoresized by SDS-PAGE and transferred onto a nitrocellulose membrane. After blocking in 5% skim-milk in PBS, the membrane was stained with PRRSV hyper immune serum at room temperature for 1 hr, followed by staining with the peroxidase-conjugated secondary Ab (goat anti-porcine IgG [H+L]; ICN Biomedicals, Inc., Aurora, OH) at room temperature for 1 hr. The presence of PRRSV proteins was determined by the addition of TMB membrane peroxidase substrate (one component) (KPL, Inc., Gaithersburg, MD). Also, the PRRSV-infected MARC-145 cell lysate was co-immunoprecipitated with PRRSV hyper immune serum. The immunocomplex was electrophoresed by SDS-PAGE and transferred onto a nitrocellulose membrane. After blocking in 5% skim-milk in PBS, the membrane was stained with anti-CD151 MAb, followed by staining with the peroxidase-conjugated secondary Ab (horse anti-mouse IgG [H+L]). The presence of CD151 bound to PRRSV proteins was determined by the addition of TMB membrane peroxidase substrate (one component).

### Virus replication assay

To investigate the effect of CD151-overexpression in MARC-145 cells, a virus replication assay was performed. The simian CD151-transfected MARC-145 cells were infected with PRRSV at 37°C for 1 hr, washed twice in MEM, and then overlaid with MEM supplemented with 1% FBS. At 18 hr postinfection, the cells were lysed by freezing and thawing, and cell debris was removed by centrifugation. The amount of virus in the supernatant was titrated by plaque assay using MARC-145 cells. In plaque assay, the supernatant was initially diluted 1:10 and in 10-fold dilutions thereafter, and used for infection to MARC-145 cells. After infection, the cells were washed twice in MEM and overlaid with MEM containing 1% FBS and 1% agar. After incubation at 37°C for 24 h, plaques were visualized by staining with 0.01% neutral red.

### Transfection of siRNA against CD151

Silencer™ pre-designed siRNA against CD151 (Ambion, Austin, TX) was used for transfection. The sequence of the siRNA strands was as follows: 5'-GUUGGAGACC

UUCAUCCAGTT-3' (sense) and 5'-CUGGAUGAAGGUCUCCAACTT-3' (antisense). The transfection of the siRNA was performed with DharmaFECT™ reagent (Dharmacon, Lafayette, CO) by following the manufacturer's instructions. MARC-145 cells were cultured overnight in a 96- or 6-well tissue culture plates. The siRNA (10 – 100 nM) was complexed with DharmaFECT™ reagent by incubating together at room temperature for 20 min. After removing the cell culture supernatant, the complex was added. After incubation for 3 days, the cells were infected with PRRSV. At 3 days post-infection, flow cytometric analysis and immunofluorescence antibody assay were performed. Flow cytometric analysis was performed as described above. For immunofluorescence antibody assay, the siRNA-transfected MARC-145 cells were fixed with 80% acetone in PBS and stained with FITC-conjugated SDOW-17 (Rural Technologies, Inc., Brookings, SD), a MAb against PRRSV nucleocapsid protein. The cells were examined by fluorescence microscopy for PRRSV.

### Checkerboard titration assay for measuring blocking activity of anti-CD151 Ab

To examine the blocking activity of anti-CD151 Ab, checkerboard titration assay was performed. MARC-145 cells were cultured overnight in a 96-well tissue culture plate (1 × 10^5 ^cells/well). The cells were incubated with PRRSV, which were pre-incubated with polyclonal anti-CD151 Ab (Santa Cruz Biotechnology, Inc., Santa Cruz, CA) or anti-β-galactosidase MAb (Boehringer Mannheim, Indianapolis, IN). The antibodies were prepared as serial two-fold dilutions starting with a 1:20 dilution, and the PRRSV preparation was initially diluted 1:10 and in 10-fold dilutions thereafter. At 2 days postinfection, the cells were fixed with cold 80% acetone at 4°C for 10 min and then incubated at 37°C for 30 min with FITC-conjugated SDOW-17, a MAb against PRRSV nucleocapsid protein. After being washed twice in PBS, the cells were examined by fluorescence microscopy.

## Competing interests

The author(s) declare that they have no competing interests.

## Authors' contributions

KS designed and carried out the experiment and drafted the manuscript.

JKK designed and carried out the experiment and drafted the manuscript.

SK designed and carried out the experiment and drafted the manuscript.

All authors read and approved the final manuscript
